# Imperforate Anus: Life Prolonged to the 102nd Day without Evacuation

**Published:** 1851-07

**Authors:** 


*Imperforate Anus: Life prolonged to the* 102*nd day without Evacuation.*—Emily Plumley was born on the 11th of June 1850; and a day or two after birth it was noticed that she was unable to pass the contents of the intestinal canal as children usually do. The parents, however, took but little notice of the fact, as one of the neighbor's children had had a similar condition of parts, and had lived comfortably for more than a month.

When the child was a month old it was admitted as a patient at the Bristol Royal Infirmary. An examination of the nates disclosed a well-formed anus, into which the point of the little finger could be passed, and through which a probe or director might be made to enter about a quarter of an inch, when it met with firm resistance. She had never passed any meconium or other contents of the intestine, but she passed water freely, took her natural food as infants ordinarily do, and had not been sick nor apparently in any way uneasy.

Upon consultation it was considered a very favorable case for an explorative puncture, to be followed up by passing a curved trocar in the suitable direction along the curve of the sacrum, for as there was a naturally-formed anus it was more than probable that the greater part of the rectum was also there. To this the mother would not consent, although the certain death of the child, if no operation was performed, was pointed out to her; and she accordingly took it away. One of the students, however, found out where she lived, and kept his eye upon the case. The child died on the 21st of September, having lived one hundred and two days, or more than three months, without ever having had an evacuation of the bowels, and having been scarcely at all sick. It never threw up anything like fæcal matter, but now and then a little of the mother's milk regurgitated, as in the case with most children. In fact, the mother stated that it was scarcely as sick as her other children were at the same age.

*Post-mortem examination, Sept.* 23.—The limbs were very thin. The abdomen much swollen; the circumference of the body measured twenty inches and a half, and it was twenty-one and a half inches in length. The organs in the chest were quite sound; the liver was of a dark purple color externally, but had a more natural appearance when cut. The stomach and first part of the small intestine were in a natural state; but about the end of the jejunum it began to be dilated, and all the lower part of the ilium was enormously distended, so as to be really longer in calibre than that of an adult; its contents were almost entirely gaseous. The cæcum was situated in the right hypochondrium, immediately below the liver. The large intestine was also much dilated, and at the lower part of the rectum measured at least four inches in diameter. At the sigmoid flexure there was a slight contraction, so that the rectum itself appeared like a large and separate pouch, and this was full of yellow and fluid fæcal matter. The muscular structure of this part of the alimentary canal was much hypertrophied, and large and distinct bands of fibre could be seen surrounding the cul de sac of the rectum. The termination below was a rounded fibrous chord, as thick as a crow quill, leading to the anus; and there was not more than a quarter of an inch between the lower part of the pouch which has been described and the highest point to which a probe could be passed through the natural aperture below. The kidneys were healthy; the bladder was distended with urine; and the ureters were very much enlarged in their canal, from the constant pressure exerted by the rectum upon the organs within the pelvis. There was a short diverticulum projecting from about the middle of the small intestine.

This case appears to me to be of considerable importance; for it shews beyond a doubt that if a child is otherwise healthy we need be in no great hurry as to operative proceedings; and that the milk of the mother is well adapted for the nutriment of the infant that although it lived for three months and never had any evacuation, the collection of fæcal matter was only sufficient to fill the distended pouch in the rectum, and probably amounted to half a pint. Of course, also, any operation that might be undertaken in such a case would be rendered easier by a larger collection of fluid contents in the intestine.—*Prov. Med. and Sur. Jour., March 5th*, 1851.

